# Drug Repositioning Discovery for Early- and Late-Stage Non-Small-Cell Lung Cancer

**DOI:** 10.1155/2014/193817

**Published:** 2014-08-18

**Authors:** Chien-Hung Huang, Peter Mu-Hsin Chang, Yong-Jie Lin, Cheng-Hsu Wang, Chi-Ying F. Huang, Ka-Lok Ng

**Affiliations:** ^1^Department of Computer Science and Information Engineering, National Formosa University, 64 Wen-Hwa Road, Hu-Wei, Yun-Lin 632, Taiwan; ^2^Division of Hematology and Oncology, Department of Medicine, Taipei Veterans General Hospital, Faculty of Medicine, National Yang-Ming University, Taipei 112, Taiwan; ^3^Cancer Center, Keelung Chang Gang Memorial Hospital, Keelung 204, Taiwan; ^4^Institute of Biopharmaceutical Sciences, National Yang-Ming University, No. 155, Section 2, Linong Street, Taipei 112, Taiwan; ^5^Genome Research Center, National Yang-Ming University, Taipei 112, Taiwan; ^6^Department of Biomedical Informatics, Asia University, 500 Lioufeng Road, Wufeng Shiang, Taichung 41354, Taiwan; ^7^Department of Medical Research, China Medical University Hospital, China Medical University, Taichung 40402, Taiwan

## Abstract

Drug repositioning is a popular approach in the pharmaceutical industry for identifying potential new uses for existing drugs and accelerating the development time. Non-small-cell lung cancer (NSCLC) is one of the leading causes of death worldwide. To reduce the biological heterogeneity effects among different individuals, both normal and cancer tissues were taken from the same patient, hence allowing pairwise testing. By comparing early- and late-stage cancer patients, we can identify stage-specific NSCLC genes. Differentially expressed genes are clustered separately to form up- and downregulated communities that are used as queries to perform enrichment analysis. The results suggest that pathways for early- and late-stage cancers are different. Sets of up- and downregulated genes were submitted to the cMap web resource to identify potential drugs. To achieve high confidence drug prediction, multiple microarray experimental results were merged by performing meta-analysis. The results of a few drug findings are supported by MTT assay or clonogenic assay data. In conclusion, we have been able to assess the potential existing drugs to identify novel anticancer drugs, which may be helpful in drug repositioning discovery for NSCLC.

## 1. Introduction

Lung cancer is the leading cause of death worldwide [1, 2]. According to medical classification, lung cancer can be divided into two major classes: small cell lung cancer (SCLC) and non-small-cell lung cancer (NSCLC). NSCLC accounts for more than 85% of all lung cancer cases, and adenocarcinoma is the most common subtype. The question of how to search for suitable potential drugs for NSCLC is an important issue in biomedical research. However, the process of new drug development is cost-intensive and time-consuming.

A previous study [3] established a systematic strategy to identify potential drugs and target genes for lung cancer. The findings from this study suggested that eight drugs from DrugBank and three drugs from NCBI could potentially reverse the expression of certain up- and downregulated genes. These results are supported by IC50 experimental data. However, the previous study can be extended in several aspects that were addressed in the present study.

Cancer is a multistage progression process that results from genetic sequences mutations, where early- and late-stage cancer-associated genes (CAG) are potentially very different. Therefore, the aim of this paper is to explore a strategy to identify stage-specific potential drugs for NSCLC through an integrated analysis of microarray profiling. In order to reduce the effect of biological heterogeneity among different individuals, normal as well as cancer tissues were taken from the same patient.

To address the target drug problem, there is a need to address the following issues. First, there is concern that different individuals may correspond to different sets of differentially expressed genes. Second, it is known that cancer is a heterogeneous disease; different stages of cancer correspond to different drug targets involving stage-specific CAG. Third, results derived from different microarray profiling vary from study to study; therefore, a rigorous approach is needed to address this problem. Fourth, reliability of drug finding prediction remains to be verified.

In order to reduce the biological heterogeneity effect among different individuals, tumor/adjacent nontumor pairwise arrays for NSCLC were employed in the present study, thus allowing pairwise statistical tests. To deal with the second issue, the samples were divided into early-stage and late-stage ones, which are denoted as stage IA/IB and stage III/IV, respectively. For the third issue, meta-analysis was adopted to integrate multiple microarray profiles. Finally, potential drug predictions were validated via biochemical assays.

Many proteins are associated with human diseases, although very often their precise functional role in disease pathogenesis remains unclear. A strategy to gain a better understanding into the interaction and function of these proteins is to make use of the protein-protein interaction (PPI) data and construct a network for disease-associated proteins. In our previous work [3, 4], it was hypothesized that the PPI networks, derived from differentially expressed genes (DEGs), could be analyzed topologically to prioritize potential drug targets.

We performed gene set enrichment analysis (GSEA) for pathway analysis and then made use of drug-gene interaction databases and the Connectivity Map (cMap) to find potential drugs for the treatment of NSCLC. It is conjectured that a small drug molecule may potentially reverse the disease signature if the molecule-induced signature is significantly negatively correlated with the disease-induced signature found in the cMap [4]. In fact, potential new treatments for cancers have been successfully identified via the cMap, including acute leukemia, colon cancer, hepatocellular carcinoma, neuroblastoma, NSCLC, and renal cell carcinoma [5–7]. Both up- and downexpressed genes are potential therapeutic targets; therefore, identification of potential drugs to treat lung cancer by using an* in silico* screening approach followed by MTT assay or clonogenic assay validation might accelerate drug discovery.

In Section 2, we give a description of the input data and the methods used in this paper. In Section 3, results for cluster analysis, enriched pathways, and cMap drug predictions are reported. We conclude in the final section.

## 2. Methods

**Figure 1:**
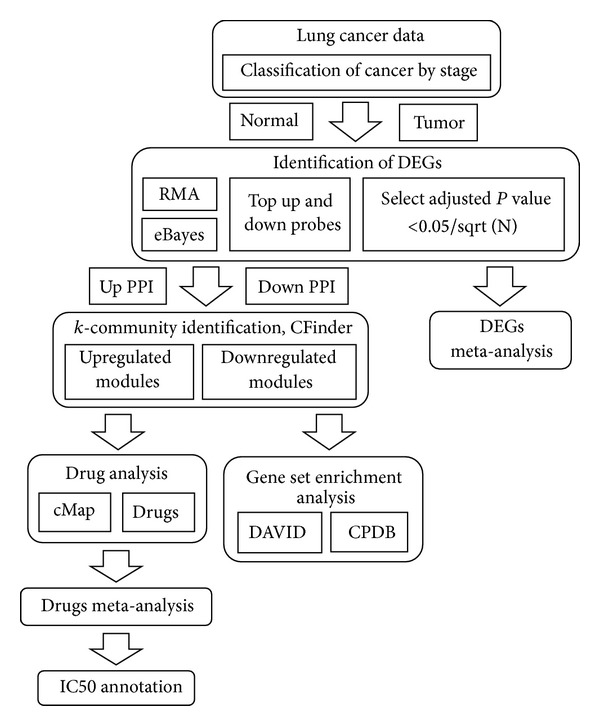
Workflow of this study.

This study proposes an* in silico* strategy to narrow down the search for lung cancer genes for target identification and drug discovery; the workflow of this study is shown in Figure 1.

### 2.1. Input Data Set

The microarray data for lung cancer was downloaded from GEO [8] and summarized in Table 1. Experiments GSE7670 [9] and GSE10072 [10] use the HG-U133A array, where GSE19804 [11] and GSE27262 [12] use HG-U133 plus 2.0 chip.

Each sample consisted of cancerous and noncancerous lung tissues obtained from a cohort of patients. To infer differentially expressed genes (DEGs), two pair tests (normal as well as cancer tissues are taken from the same patient) were conducted. The main advantage of using paired samples is that it could reduce the biological heterogeneity effect. In the late stages of cancer, it is very common to find cell invasion, metastasis, and drug-resistance related genes [13]. To investigate this issue, we divided the samples into early- and late-stage ones. Early-stage samples were taken from patients with stage I, IA, and IB cancers, whereas late-stage data were obtained from stage III and IV patients.

### 2.2. Microarray Data Analysis

Microarray technology allows for high-throughput screening and analysis of tens of thousands of genes at the same time. Some genes are activated or inhibited, and some are DEGs, which due to certain regulatory factors, result in changes in gene expression levels by a few times, ten times, or more. Given sets of microarray data, one can identify DEGs among a large number of gene expressions and understand the mechanism of lung cancer formation induced by these DEGs.

There are many microarray data analysis methods, such as using the concept of false discovery rate (FDR) to screen for significant genes [14], using ANOVA to explore the impact of microarray gene expression values within a single factor [15], and clustering analysis. Among the many statistical methods, significance analysis of microarray (SAM) [16, 17], empirical Bayes analysis of microarrays (EBAM) [18], and empirical Bayes statistics (eBayes) [19] are three commonly employed approaches to screen DEGs. The publicly available microarray data analysis package* Bioconductor* [20, 21] was adopted to perform such calculations.

The statistical method eBayes was chosen in this study because it was found that eBayes, SAM, and EBAM achieve a similar level of cancer gene prediction accuracy [22]. The selected DEGs were divided into two groups, an upregulated group (up probes in Figure 1) and a downregulated group (down probes), according to the gene expression fold change (FC) values.

Among the DEGs, genes were classified as either up- or downregulated genes if the log⁡_2_⁡FC was less than or greater than zero, respectively. Any gene expression level with fold change less than 5.64 (log⁡_2_⁡50) was reset to 5.64 in order to facilitate the cMap search.

### 2.3. Cluster Analysis

We adopted BioGrid version 3.2.101 in our analysis, which consists of 209,838 PPI records. In a PPI network, a densely connected area is referred to as a cluster, which is a functional module. Nodes having a high degree of connection are defined as hubs and are more likely to be essential. Members of a cluster are usually involved in similar biological processes, and protein complexes can be identified through the clustering of a network [23, 24]. It is suggested that a protein complex is a biologically functional module composed of subunits performing similar functions [25]. Given two proteins, *A* and *B*, with a PPI, if both *A* and *B* are obtained from the eBayes prediction as upregulated, then the PPI among *A* and *B* is the so-called up PPI. Communities constructed from up PPI are called upregulated communities.

To investigate the functional modules in which potential lung cancer related proteins are involved, a set of highly confident human PPIs were input to the CFinder software [26] to perform an analysis based on the clique percolation clustering approach [27]. A *k*-community was set with *k* being equal to three (complete subgraphs of size *k*). Any two *k*-communities are adjacent if they share *k* − 1 common nodes. A *k*-community (*k* ≥ 4) is constructed by merging all possible adjacent (*k* − 1)-communities.

### 2.4. Gene Set Enrichment Analysis (GSEA)

DAVID [28] is a web-based resource which provides batch annotation and GO [29] term enrichment analysis to highlight the most relevant GO terms associated with a given gene list. The ConsensusPathDB (CPDB) [30] tool provides gene set analysis and metabolite set analysis. The DAVID tool is based on the Fisher exact test, while the CPDB tool is based on the Wilcoxon test. To find the enriched pathways of our lung cancer gene signature, we performed an overrepresentation pathway analysis using both DAVID and CPDB. Under the threshold of a *P* value of less than 0.005, enriched pathways from the overrepresentation analysis including up- and downregulated *k*-communities were obtained from CFinder analysis. Significant pathway results were ranked according to the *P* value. Thus, enriched GO terms for these two protein groups were obtained. We used both tools in this stage for cross-verification.

### 2.5. Potential Target Genes and Drug Discovery

Both of the up- and downregulated communities are derived from the CFinder tool and were used to query the cMap database, where potential drugs with *P* values of less than 0.05 are retained. To identify target genes, the FDA-approved drugs and the chemical-protein links data from STITCH [31] were merged. The Gene Name Service was then used to translate the protein ID to its corresponding HUGO-approved gene symbol and Entrez gene ID. Drugs obtained from the cMap output were mapped and finally identified with known drug targets in the cancer up- or downregulated PPI network.

The idea of drug repositioning is a recently developed approach in the pharmaceutical industry that endeavors to identify new uses for existing drugs and has achieved certain successes [32]. Furthermore, this approach has the potential to accelerate the development time for drugs, as well as reducing side effects. There are many works on identifying repositioned drugs, which are based on various methods: the graph-based inference method [33, 34], the microarray expression method [35], the differential expressed correlation method [36], and the integration of phenotypic, chemical indexes and PPI method [37], and using the drug-gene-disease relationship [38]. We also note that CancerResource [39] is a very comprehensive resource for drug repositioning study.

Several issues arise from combining different datasets, such as the problem of data heterogeneity, different sample sizes, and the data dependence problem. In principle, these issues can be tackled by employing a meta-analysis approach. Meta-analysis (MA) [40, 41] is a set of statistical methods for summarizing the results of several studies into a single estimate. The strength of MA is that it is capable of identifying relationships across a number of different studies.

For the drug prediction study, cMap provides an enrichment score, *ρ*, and a *P* value to quantify each cMap drug. The *ρ* value lies between −1 and 1; therefore it can be treated as a sample correlation coefficient and serve as an effect size index for MA [41]. In practice, *ρ* is transformed to the Fisher *z* scale, and all the analyses are conducted using the converted values. After the analyses are completed, the *z* values are transformed back to the original metric. The transformation to Fisher's *z* is given by

(1) $$\begin{array}{c} z=\frac{1}{2}\ln\frac{1+\rho }{1-\rho } \end{array}$$

and the variance of *z* is defined by *V*_*z*_ = 1/(*N* − 3), where *N* denotes the sample size.

The weight assigned to each study in a fixed-effect model is given by

(2) $$\begin{array}{c} W_{i}=\frac{1}{V_{Y_{i}}}, \end{array}$$

where *W*_*i*_ is the within-study variance for study *i*. The weighted mean (*M*) is computed as

(3) $$\begin{array}{c} M=\frac{\sum_{i=1}^{k}W_{i}Y_{i}}{\sum_{i=1}^{k}W_{i}}. \end{array}$$

For unweighted calculations, *W*_*i*_ equals one. The variance of the summary effect (*V*_*M*_ ) is given by

(4) $$\begin{array}{c} V_{M}={\left(\overset{k}{\underset{i=1}{\sum }}W_{i}\right)}^{-1}. \end{array}$$

For unweighted calculations, the *Z*-score for normal distribution is defined by

(5) $$\begin{array}{c} Z=\frac{M}{{\text{SE}}_{M}}, \end{array}$$

where SE_*M*_ denotes the standard error and is equal to $\sqrt{V_{M}}$.

For weighted calculations, the *Z*-score is defined by

(6) $$\begin{array}{c} Z=\frac{\sum_{i=1}^{k}W_{i}Y_{i}}{\sqrt{\sum_{i=1}^{k}W_{i}^{2}}}. \end{array}$$

From (7), one can determine the one-tailed test *P* value.

The 95% lower and upper limits for the summary effect would be computed as

(7) LLM=M−1.96×SEM,ULM=M+1.96×SEM.

The formula for the random-effects model is given in a monograph written by Borenstein et al. [41]. The above analyses allow us to determine the confidence interval of the CC, *r*.

Besides the use of *ρ*, the use of the Fisher combined test (FCT) [40] is another option. The Fisher summary statistic method combines the *P* values and is defined by

(8) $$\begin{array}{c} F_{i}=-2\overset{N}{\underset{j=1}{\sum }}\log\left(p_{ij}\right) \end{array}$$

which tests (chi-square *χ* ^2^) the null hypothesis for gene *i*, where indices *i* and *j* denote the *i*th gene from the *j*th dataset, respectively. However, cMap may return a zero *P* value, hence rendering (8) infinite; therefore, it was not used in the present analysis.

There are two models in meta-analysis: the fixed-effect model and random-effect model [41]. In the fixed-effect model it is assumed that there is only one true effect size and that all differences among the studies or batches are due to sampling errors only. In contrast, the random-effect model allows the effect size to vary from study to study. Each study estimates a different effect size. These two models are considered in our work.

In other words, a test for homogeneity of distribution was performed. As it is rather common to find that the effect size may vary from one study to the next, we employ the MA method, such as the *Q* statistics and *I* ^2^ statistics, to quantify the heterogeneity, to test it, and to incorporate it into the weighting scheme. We use a *P* value of 0.1 for *I* ^2^ statistics as the criterion for statistical significance. A *P* value larger than or equal to 0.1 means that there is little variation between batches; then a fixed-effect model might be appropriate; otherwise choose random-effect model [41]. Degree of heterogeneity is characterized by the *I* ^2^ value. A value of *I* ^2^ less than 25% implies no heterogeneity, whereas a value larger than 75% means extremely high heterogeneity.

If the studies are homogenous, then it is likely that the various studies are testing the same hypothesis. If these estimates are heterogeneous, then it is probable that each study is not testing the same hypothesis. Therefore, it may not be appropriate to combine all the study results into one meta-analysis. In such case, we would need to conduct a separate meta-analysis, such as meta-regression analysis for different subsets of studies [41].

### 2.6. Cell Culture

All cell-culture-related reagents were purchased from Invitrogen. Human lung cancer cell lines A549 and H460 were purchased from the American Type Culture Collection/Bioresource Collection and Research Center (BCRC) (Taiwan). These cells have performed STR-PCR profile at BCRC. A14 was a derivative of A549 cells stably selected with a p53 shRNA construct. Human lung adenocarcinoma cell lines, CL_1-0_ and CL_1–5_, were kind gifts from Dr. Pan-Chyr Yang. H1299 stable clones (transfected with EGFR-WT (wild type) and EGFR-L858R mutant) were kindly provided by Dr. Yi-Rong Chen. All cells were cultured in RPMI 1640 with 10% fetal bovine serum (FBS), 2 mM of L-glutamine, and 1% penicillin/streptomycin and maintained in a 37°C, 5% CO_2_ incubator.

### 2.7. MTT Cell Viability Test

Cell viability was determined using an MTT assay. Cells were seeded in a 96-well microplate for 16~20 hrs and treated with the indicated drugs. After drug treatment for 72 hrs, 50 *μ*L MTT solution (2 mg/mL) per well was added and incubated at 37°C. Two hours later, 150 *μ*L liquid per well was removed and DMSO was added and the absorbance at 570 nm was detected using an ELISA reader (Infinite M1000, TECAN, Switzerland). The untreated groups were considered to be 100% viable.

### 2.8. Clonogenic Assay

Two NSCLC cell lines, A549 and H460, were seeded in 6-well plates with 500 cells/well for 7–10 days. Each well contained 1.5 mL RPMI medium as culture condition and tested drugs were added 24 hrs after the seeding of the cells. The medium and drugs were changed once on day four. After treatment, cells were washed with PBS, and the colonies were fixed (acetic acid : methanol, 1 : 3) and stained with 0.5% crystal violet in methanol. After removing the excess crystal violet and rinsing with tap water, the colonies were counted manually.

## 3. Results

### 3.1. Microarray Data Analysis

In this study multiple microarray source data were employed for analysis. Robust multiarray average (RMA) was used for gene expression normalization. The eBayes analysis was subsequently conducted on the previous results. DEGs were predicted by an eBayes with an adjusted *P* value of 0.005. By integrating with the BioGrid [42] PPI data, a list of binary interactions among DEGs was determined for the up and down groups.

There may be concern regarding the use of different microarray platforms being subjected to heterogeneity problem. We note that the following two steps can tackle such concern: (i) selecting common DEGs among all the platforms for further analysis and (ii) employing meta-analysis approach and performing test of heterogeneity to determine whether the fixed-effect model or random-effect model is needed.

A total of 642 and 780 genes were identified as the common DEGs for the early- and late-stage cancer, respectively. The results of the total number of DEGs, “UP” and “DOWN” DEGs for early- and late-stages of cancer, are reported in Table 2. The second last column in the table denotes the total number of UP and DOWN DEGs for different GSE platforms. It is noted that the number of “DOWN” DEGs identified is larger than “UP” DEGs in both of early- and late-stages, in which the ratio is about 2 to 1.

### 3.2. Cluster Analysis

Genes which do not highly interact with other genes are assumed to be less important and consequently such genes were removed before the subsequent analysis. Hence, by CFinder, any gene which did not belong to a *k*-community was excluded. We also counted the number of *k*-communities in the NSCLC PPI network and found there was no community with *k* larger than five.  Table 3 summarizes the number of *k*-communities identified by CFinder. For early-stage, a total of six and sixteen genes belong to the two up- and seven downregulated *k*-communities, respectively, whereas a total of forty-five and sixteen genes belong to the thirty-four up- and six downregulated *k*-communities, respectively, for late-stage.

Only genes belonging to the communities identified by CFinder were selected for the next stage of analysis.

### 3.3. Enriched Biological Pathways

Pathway annotation of communities was given by implementing DAVID and CPDB. According to REACTOME [43] and KEGG [44] databases, pathways with their *P* values less than 0.05 and ranked among the top ten are reported. Using the annotation tool in DAVID database REACTOME, Table 4 lists the enriched pathways information for early- and late-stage NSCLC. The “Count” and “%” columns denote the number of overlapped genes in the filtered community genes and the corresponding pathway and the percentage of overlapped genes, respectively. As we noted from Table 4, GSEA suggested that hemostasis, signaling in immune system, integrin cell surface interaction, and metabolism of carbohydrates are enriched pathways for early-stage cancer, whereas cell cycle and DNA replication pathways are ranked among the top for late-stage cancer. It is noted that these late-stage cancer pathways are dominated by cell-cycle related processes.

Cancer is a multistage progression process that results from mutations in genetic sequences. Accumulation of genetic mutations could lead to a defective DNA repair mechanism, consequently giving rise to genetic instability and uncontrolled cell growth [45].

Numerous studies have reported that homeostasis and cancer formation are related [46–49]. Integrins are the receptors that mediate cell adhesion to the extracellular matrix (ECM). Varner and Cheresh [50] pointed out in 1996 that ECM receptors, integrins, regulate the cellular proliferation machinery in tumor cells. In the seminal review paper written by Hanahan and Weinberg [51], it was reported that integrin can influence cell behavior and transform cells into an active proliferative state. Recent studies have also suggested that integrins are involved in cancer progression [52, 53] and lung squamous cell carcinoma [54]. Furthermore, elevated glucose consumption is observed in tumor formation [55–57].

Late-stage cancer patients commonly have cell invasion and metastasis. Malignant cells have the ability to invade adjacent normal tissue structures. Malignant tumor cells break off from the tumor and enter blood vessels or the lymphatic system and migrate to other parts of the body and initiate another tumor. Biomedical studies have suggested that the development of the metastatic process involves an interaction between cell cycle signaling, adhesion pathways, and epithelial-mesenchymal transition program [13]. It is also known that signal transduction pathways, such as p53, MAPK, Notch, and ROS, are heavily involved in metastasis [58]. In particular, mutations in p53 and K-RAS appears only later in tumor progression [45].

Defects in the cell cycle mitotic checkpoint generate aneuploidy and might facilitate tumorigenesis [59]. Mitotic progression and sister-chromatid segregation are controlled by the anaphase promoting complex/cyclosome (APC/C). APC/C forms a protein complex with its mitotic coactivator, CDC20, which controls mitotic progression [59]. CDC20 protein level may directly affect cell fate during prolonged mitotic arrest [60] and its turnover rate may be a key factor in cancer patient response to antimitotic therapies [61].

Using the CPDB tool, the top ten most significant pathways for early-stage NSCLC and late-stage NSCLC returned by REACTOME are listed in Table 5. Again, GSEA suggested that hemostasis, cell surface interaction, and metabolism of carbohydrates, that is, glycolysis and gluconeogenesis, are the enriched pathways for early-stage cancer. For late-stage cancer, again it is found that the cell cycle pathways are ranked among the top pathways. Essentially, results returned by DAVID and CPDB are consistent with each other.

From Table 5, it is found that PECAM1 [62–64] and CBL are frequently altered in lung cancer [65], and CD28 is associated with NSCLC formation [66]. PECAM1 interactions are related to angiogenesis.

Using the KEGG database, pathways with *P* value less than 0.05 returned by DAVID are listed for early-stage and late-stage cancer in Table 6. Again, enrichment analysis suggested that glycolysis/gluconeogenesis and cell signaling are the enriched pathways for early-stage cancer. It is known that integrin is a key regulator of cell adhesion [53].

It was also found that the cell cycle pathway and DNA replication pathway were ranked among the top pathways for late-stage cancer. It is known that cancer is due to uncontrolled cell mitosis, and this uncontrolled process is a common element in all types of cancer.

Cell adhesion molecules (CAMs), a diverse system of glycoproteins, have been found to play an important role in cancer progression and in the application of cancer therapy [67–69]. Tight junctions are cellular structures located at the apicobasal region of epithelial cell membranes [70]. It has been experimentally found that lung tumors show changes in the expression in tight junction proteins [71]. Other studies have also indicated that tight junction proteins show aberrant expression in breast cancer [72] and correlate with metastasis [73–75].

We noted that the significant enriched pathways found in Table 6 (late-stage) are also identified in the work by Liu et al. [76]. Except oocyte meiosis, the other four pathways are involved in two NSCLC subtypes: adenocarcinoma and squamous cell carcinoma.

Using the CPDB tool, significant pathways returned by KEGG are listed in Table 7. Again it was found that the cell cycle pathway and DNA replication pathway are ranked among the top pathways for late-stage cancer.

The hypoxia-inducible factor-1 (HIF-1) is an oxygen-sensitive transcriptional activator and is causally involved in NSCLC [77–79].

Again, the cell cycle pathway ranked first (among the top of the list) both in REACTOME and KEGG using CPDB. In other words, analyses using DAVID and CPDB are in good agreement. Relative to DAVID, CPDB tends to return more pathway information.

Integrins are the receptors that mediate cell adhesion to ECM. The extracellular matrix (ECM) is a network of macromolecules that underlies all epithelia and endothelia and that surrounds all connective tissue cells. This matrix provides mechanical strength and also influences the behavior and differentiation state of cells in contact with it.

### 3.4. Potential Drugs and Their Target Genes for NSCLC

Both the up- and downregulated communities extracted from CFinder were analyzed by cMap. Under the constraint of an enrichment score (ES) of less than zero, and cMap drugs associated with *P* value, that is, cMap *P* value less than 0.1 or 0.5, potential drugs were inferred by performing MA. Fisher's summary statistic method was used for combining cMap *P* value.

After performing meta-analysis using ES as the effect size, twenty-four potential drugs were found with a *P* value for MA being less than 0.05 for early-stage cancer. The results are listed in Table 8. Among the twenty-four drugs, two drugs tested by MTT or clonogenic assay were validated as effective (i.e., mebendazole and prenylamine).

From Table 8, among the 30 potential drugs (*P* value for MA is less than 0.05) for late-stage cancer, there were six drugs tested by MTT or clonogenic assay and validated as effective, that is, mebendazole, spiperone, anisomycin, pyrvinium, mefloquine, and niclosamide.

We performed the heterogeneity test on the 24 drugs for early-stage and the 30 drugs for the late-stage cancer using the *I* ^2^ statistics. It is found that both of the fix-effect model and the random-effect model are required according to the *I* ^2^ statistics test with a *P* value less than 0.1 [41].

We used available drugs in the list for* in vitro* cytotoxic validations (Table 9). Certain drugs showed effective cytotoxic effects for lung cancer cells. However, the very limited data showed that there were inconsistencies in MTT and clonogenic assays. For example, mebendazole showed a good IC50 in MTT (<1 *μ*M) but not in the clonogenic assay (>10 *μ*M). On the other hand, spiperone showed a relatively effective IC50 (<10 *μ*M) in clonogenic assay rather than in MTT assay (>10 *μ*M). This phenomenon is still hard to explain in the current status.

**Figure 2:**
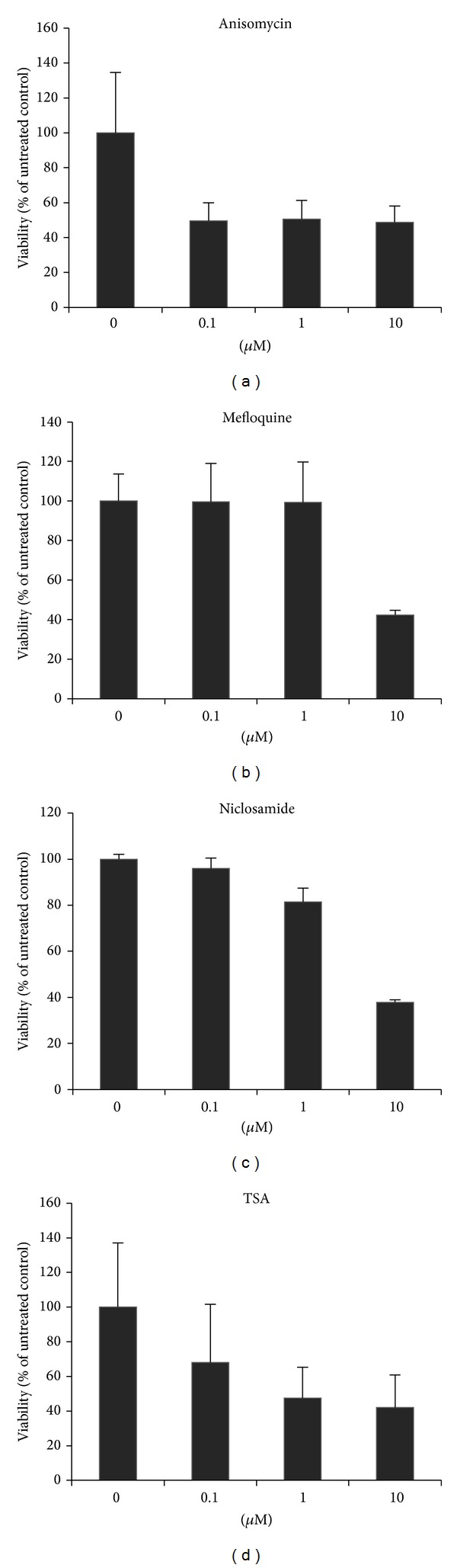
Dose-dependent plots for four of the representable drugs, that is, anisomycin, mefloquine, niclosamide, and trichostatin A (TSA).

Dose-dependent figures for four of the representable drugs are shown in Figure 2. The reasons to use two most commonly used lung cancer cell lines A549 and H460 include the following: (i) they have different histologic subtypes, that is, A549 is adenocarcinoma and H460 is large cell carcinoma, although both belong to non-small-cell carcinoma; (ii) the origin of A549 cell was obtained from lung tissue and H460 was from lung pleural effusion, which may represent different stages of lung cancer; and (iii) both cells are EGFR wild type that could be tested by the drugs potentially effective for intrinsic EGFR-TKI resistance.

We conducted meta-analysis using the *P* values from cMap drugs obtained from individual arrays. As shown in Table 10, the first row lists the early- and late-stage ES and *P* value used for meta-analysis. Entries in the lower diagonal denote the number of common drugs for MA choosing ES and *P* value as the effective sizes, and, in contrast, entries in upper-diagonal show its Jaccard index (JI) score. Given two sets *A* and *B*, JI(*A*, *B*) is defined as |*A*∩*B* | /(|*A* | ∪|*B* | −|*A*∩*B*|), where |*A*∩*B*|, |*A*| and |*B*| denote the cardinality of *A*∩*B*, *A* and *B*, respectively.

For early-stage cancer, there are five common drugs (JI is 0.156) predicted by both ES and cMap *P* value meta-analysis, whereas there are ten common drugs (JI is 0.217) for late-stage cancer. The number of common drugs for both early- and late-stage cancer are around five or six, assuming ES versus *P* value. There are sixteen (JI = 0.421) and six (JI = 0.182) common drugs predicted by ES and *P* value meta-analysis, respectively. This seems to indicate that MA tends to return a higher overlapping between early- and late-stage results.

We submitted the selected drugs to DrugBank and NCBI to search for up- and downregulated target genes. The results of the number of target genes are summarized in Table 11, which are potential therapeutic targets for future lung cancer clinical trials. For early-stage cancer, no target gene is reported by using the GSE7670 platform; therefore, we report common drug target genes among the rest of the other three microarray platforms. For late-stage cancer, we report common drug target genes among any two of the three platforms.

Table 12 summarizes the up- and down communities' drug target genes. As it is shown in the table, certain genes are predicted by both effect size studies. For instance, up community genes, RPL26L1, FEN1, and IDH1, are found in both studies.

**Figure 3:**
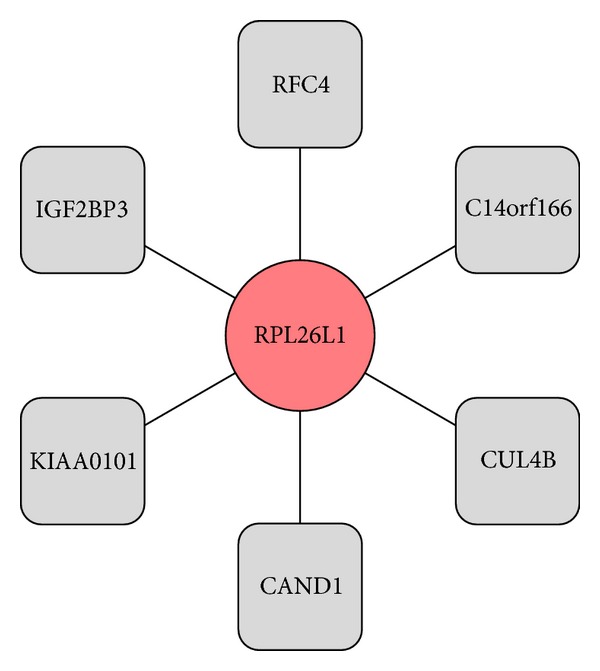
The upregulated target genes (circles) PPI partners (squares); solid line represents PPI.

**Figure 4:**
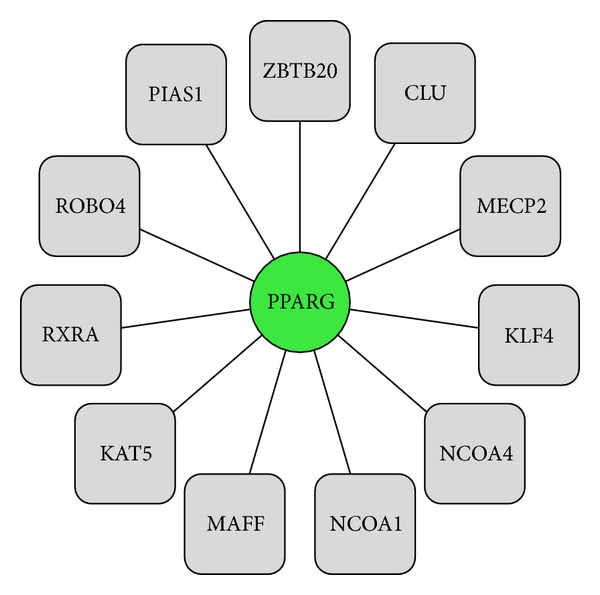
The downregulated target genes (circles) PPI partners (squares); solid line represents PPI.

Figure 3 depicts the PPI network of upregulated target genes using Cytoscape [80]. The upregulated target gene RPL26L1 directly interacts with six proteins; Figure 4 represents the PPI network of downregulated target gene, PPARG. This gene directly interacts with eleven proteins.

## 4. Conclusion

We applied the meta-analysis technique to infer therapeutic drugs for NSCLC treatment by integrating microarray expression profiles. Since cancer is a multistage progressive disease, early- and late-stage CAG are potentially very different; therefore, stage-specific DEGs were identified. PPI data were then employed to construct dense PPI modules. The up- and downregulated communities were used as queries to perform functional enrichment analysis and potential drug identification using cMap. Drugs can act on not merely the transcription level, but rather on the protein, posttranscription, or posttranslation levels. Large-scale drug screening needs fast and efficient ways. In the current status, using gene expression change to infer drug repositioning is the most suitable way, which has been claimed in the rationale of cMap original paper [81]. It is still difficult to see the modulations of protein level in such a large-scale, high throughput method.

Enrichment analysis suggests pathways that are early- and late-stage specific. This supports the use of the meta-analysis technique to derive reliable results when combining multiple gene expression datasets.

Enrichment scores and *P* values obtained from cMap were adopted as the effect size indices for target drug meta-analysis. Certain common drugs were found by using the enrichment score and *P* value meta-analysis technique. A fraction of our drug findings results are supported by IC50 experimental data.

Our findings suggest that certain up- and downregulated genes are potential drug targets. Furthermore, the drugs derived from DrugBank and NCBI are potential lung cancer therapeutic drugs.

In summary, we have developed a pipeline to infer therapeutic drugs for disease treatment by integrating microarray, PPI, and the cMap resources. Meta-analysis was adopted to integrate multiple datasets. Up- and downregulated communities were used as queries to perform functional enrichment analysis and potential drug prediction. Overrepresented cancer stage-specific pathways are determined. The target drug results are supported by IC50 measurement data. It is expected that the approach developed in the current work should be of value for future studies into understanding the molecular mechanism of lung cancer formation and identifying therapeutic drug targets.

## Figures and Tables

**Table 1:** Summary of microarray datasets.

GEO ID	Organization name	Number of samples (early-stage)	Number of samples (late-stage)
GSE7670	Taipei Veterans General Hospital	8	11
GSE10072	National Cancer Institute, NIH	15	9
GSE19804	National Taiwan University	35	13
GSE27262	National Yang-Ming University	25	n/a

**Table 2:** Statistics of DEGs for early- and late-stage NSCLC.

Early-stage NSCLC						
	GSE7670	GSE10072	GSE19804	GSE27262	Common DEGs	^ #^PPI
DEGs	642	642	642	642		
UP DEGs	213	213	214	212	211	41
DOWN DEGs	429	429	428	430	426	105

Late-stage NSCLC						
	GSE7670	GSE10072	GSE19804	GSE27262	Common DEGs	^ #^PPI

DEGs	780	780	780	n/a		
UP DEGs	257	258	257	n/a	254	166
DOWN DEGs	523	522	523	n/a	520	133

^#^PPI denotes the total number of PPI among common DEGs.

**Table 3:** Total number of *k*-communities identified by CFinder.

Early-stage NSCLC				
*k*	Up group	Number of genes	Down group	Number of genes
3	2	6	6	12
4	0	0	1	4

Late-stage NSCLC				
*k*	Up group	Number of genes	Down group	Number of genes

3	31	37	6	16
4	3	8	0	0

**Table 4:** Summary of pathways returned by REACTOME using DAVID for early- and late-stage NSCLC.

Early-stage NSCLC			
Term	Count	%	*P* value
Hemostasis	6	27.3	2.87*E* − 04
Signaling in immune system	6	27.3	7.22*E* − 04
Integrin cell surface interactions	3	13.6	0.0223
Metabolism of carbohydrates	3	13.6	0.0295

Late-stage NSCLC			
Term	Count	%	*P* value

Cell cycle, mitotic	21	34.4	8.49*E* − 12
Cell cycle checkpoints	10	16.4	3.08*E* − 06
Cdc20: phospho-APC/C-mediated degradation of cyclin A	6	9.8	8.20*E* − 04
APC-Cdc20-mediated degradation of Nek2A	4	6.6	0.00211
DNA replication	5	8.2	0.0239

**Table 5:** Summary of the top ten pathways returned by REACTOME using CPDB for early- and late-stage NSCLC.

Early-stage NSCLC			
Pathway name	Effective size	% of overlap	*P* value
Cell surface interactions at the vascular wall	94	6.4%	1.53*E* − 08
Nephrin interactions	23	13%	1.02*E* − 05
Hemostasis	463	1.5%	1.45*E* − 05
Glycolysis	28	10.7%	1.87*E* − 05
Gluconeogenesis	32	9.4%	2.82*E* − 05
PECAM1 interactions	12	16.7%	0.00022
Integrin cell surface interactions	66	4.5%	0.00025
Glucose metabolism	67	4.5%	0.00026
Regulation of signaling by CBL	18	11.1%	0.00052
CD28 dependent PI3K/Akt signaling	21	9.5%	0.00071

Late-stage NSCLC			
Pathway name	Effective size	% of overlap	*P* value

Cell cycle	442	5.9%	5.15*E* − 21
Cell cycle, mitotic	355	6.8%	1.02*E* − 20
Mitotic M-M/G1 phases	214	8.4%	3.88*E* − 17
APC/C-mediated degradation of cell cycle proteins	38	26.3%	4.87*E* − 15
Regulation of mitotic cell cycle	38	26.3%	4.87*E* − 15
M phase	183	8.2%	3.57*E* − 14
Mitotic prometaphase	110	10.9%	5.59*E* − 13
Mitotic anaphase	130	9.2%	4.22*E* − 12
Mitotic metaphase and anaphase	131	9.2%	4.63*E* − 12
Resolution of sister chromatid cohesion	101	10.9%	5.80*E* − 12

**Table 6:** Summary of top pathways returned by KEGG using DAVID for early- and late-stage cancer.

Early-stage NSCLC			
Term	Count	%	*P* value
Leukocyte transendothelial migration	4	18.2	0.00294
Glycolysis/gluconeogenesis	3	13.6	0.00982
Epithelial cell signaling in *Helicobacter pylori* infection	3	13.6	0.0125
Cell adhesion molecules (CAMs)	3	13.6	0.0432
Tight junction	3	13.6	0.0444
Focal adhesion	3	13.6	0.0911

Late-stage NSCLC			
Term	Count	%	*P* value

Cell cycle	12	19.7	3.22*E* − 10
Oocyte meiosis	8	13.1	7.47*E* − 06
Progesterone-mediated oocyte maturation	6	9.8	2.68*E* − 04
DNA replication	3	4.9	0.0250
p53 signaling pathway	3	4.9	0.0791

**Table 7:** Summary of top pathways returned by KEGG using CPDB for early- and late-stage cancer.

Early-stage NSCLC			
Term	Effective size	% of overlap	*P* value
Leukocyte transendothelial migration	118	3.4%	6.37*E* − 05
Glycolysis/gluconeogenesis	66	4.5%	0.000250
Epithelial cell signaling in *Helicobacter pylori* infection	68	4.4%	0.000273
HIF-1 signaling pathway	106	2.8%	0.00100
Tight junction	134	2.2%	0.00197
Cell adhesion molecules (CAMs)	147	2.0%	0.00257

Late-stage NSCLC			
Term	Effective size	% of overlap	*P* value

Cell cycle	124	9.7%	2.39*E* − 12
Oocyte meiosis	110	7.3%	1.38*E* − 07
Progesterone-mediated oocyte maturation	86	7.0%	7.25*E* − 06
DNA replication	36	8.3%	0.00100
Epstein-Barr virus infection	203	2.5%	0.00512
Viral carcinogenesis	206	2.4%	0.00544
p53 signaling pathway	68	4.4%	0.00620
Measles	134	3.0%	0.00630
Hepatitis B	146	2.7%	0.00849

**Table 8:** The number of IC50 verified drugs and potential drugs identified by using ES, cMap *P* value less than 0.1 and 0.5 for early- and late-stage NSCLC.

Effect size	Early-stage	Late-stage
ES	2/24	6/30
cMap *P* value < 0.1	3/13	5/26
cMap *P* value < 0.5	8/56	7/65

Numbers before and after the slash sign (/) denote the numbers of IC50 verified drugs and potential drugs, respectively.

**Table 9:** IC50 values of potential drugs for early- and late-stage NSCLC.

Effect size	Stage	cMap drug name	MTT	Clonogenic
ES	Early	Mebendazole	<1	>10
		Prenylamine	>5	>10
	Late	Mebendazole	<1	>10
		Spiperone	>10	<10
		Anisomycin	<0.1	
		Pyrvinium	<0.1	
		Mefloquine	>5	
		Niclosamide	>5	

*P* value < 0.1	Early	Trichostatin A	<1	
		Monensin	<1	
		Cloperastine	<10	
	Late	Trichostatin A	<1	
		Mefloquine	>5	
		Pyrvinium	<0.1	
		Securinine	>5	
		Nortriptyline	<10	

**Table 10:** The number of common drugs for early-stage and late-stage using the enrichment score (ES) and cMap *P* value (less than 0.1) for meta-analysis.

Effect size	ES		*P* value	
	Early-stage	Late-stage	Early-stage	Late-stage
ES				
Early-stage		0.421	0.156	0.136
Late-stage	16		0.132	0.217
*P* value < 0.1				
Early-stage	5	5		0.182
Late-stage	6	10	6	

**Table 11:** The results of up- and downregulated target genes within *k*-communities obtained from DrugBank and NCBI.

	GSE7670	GSE10072	GSE19804	GSE27262
Early-stage				
Up	0	7	10	15
Down	0	5	7	14

Late-stage				
Up	1	4	7	n/a
Down	2	1	7	n/a

**Table 12:** The results of up- and downregulated target genes within *k*-communities using ES and cMap *P* value as the effect size.

Effect size		
ES		
Early-stage	Up-community gene	Down-community gene
	FEN1, IDH1, PSMB2, PSMB5, RPL26L1	NR3C1, PPARG
Late-stage	PSMB2	NR3C1, PPARG
*P* value < 0.1		
Early-stage	EZH2, FEN1, IDH1, and RPL26L1	NR3C1, PPARG
Late-stage	Not available	NR3C1, PPARG
